# Lactate Modulates Cellular Metabolism Through Histone Lactylation-Mediated Gene Expression in Non-Small Cell Lung Cancer

**DOI:** 10.3389/fonc.2021.647559

**Published:** 2021-06-02

**Authors:** Jun Jiang, DengLiang Huang, Yuan Jiang, Jing Hou, MeiYuan Tian, JianHua Li, Li Sun, YaoGang Zhang, Tao Zhang, ZhiQin Li, ZhongCheng Li, SiXian Tong, YanYan Ma

**Affiliations:** ^1^Oncology Department, Affiliated Hospital of Qinghai University, Xining, China; ^2^Central Laboratory, Affiliated Hospital of Qinghai University, Xining, China; ^3^Qinghai Province Research Key Laboratory of Echinococcosis, Affiliated Hospital of Qinghai University, Xining, China; ^4^Rehabilitation Department, Affiliated Hospital of Qinghai University, Xining, China; ^5^Department of Scientific Research Office, Affiliated Hospital of Qinghai University, Xining, China

**Keywords:** lactate, lactylation, metabolism, gene expression, non-small cell lung cancer

## Abstract

Lactate has been observed to fuel TCA cycle and is associated with cancer progression in human lung cancer, the leading cause of cancer deaths worldwide, but the effect of lactate on lung cancer metabolism is rarely reported. In this study, disordered metabolism in non-small cell lung cancer was demonstrated by increased G6PD and SDHA protein levels *via* immunofluorescence, and up-regulated lactate dehydrogenase was found to be associated with poor prognosis. Then flow cytometry and Seahorse XFe analyzer were utilized to detect the effect of lactate on glycolysis and mitochondrial function in non-small cell lung cancer cells. The results show that in non-small cell lung cancer cells lactate attenuates glucose uptake and glycolysis while maintaining mitochondrial homeostasis as indicated by improved mitochondrial membrane potential. Further exploration found that mRNA levels of glycolytic enzymes (*HK-1*, *PKM*) and TCA cycle enzymes (*SDHA*, *IDH3G*) are respectively down-regulated and up-regulated by lactate, and increased histone lactylation was observed in promoters of *HK-1* and *IDH3G via* chromatin immunoprecipitation assay. Taken together, the above results indicate that lactate modulates cellular metabolism at least in part through histone lactylation-mediated gene expression in non-small cell lung cancer.

## Introduction

Lung cancer has been reported as the most commonly diagnosed cancer and the leading cause of cancer deaths both in China and worldwide in two sexes combined. It was estimated that lung cancer accounted for 11.6% of 18.1 million new cancer cases and 18.4% of 9.6 million cancer deaths globally in 2018 (1), and 787 thousand diagnoses of lung cancer with 631 thousand lung cancer deaths were estimated among Chinese population in 2015 (2). The two major histological forms of lung cancer are small-cell lung cancer (SCLC) and non-small-cell lung cancer (NSCLC), respectively making of about 85% and 15% of all lung cancer cases (3). Though significant progression has been achieved in targeted therapy and immunotherapy for lung cancer treatment, prognosis is still dismal, with a 5-year survival rate just being less than 17% (4).

Metabolic reprogramming is a hallmark of cancer cells (5). In addition to the common genetic alterations, such as mutations in *TP53*, *EGFR*, *KRAS*, and rearrangements in *RET*, *ROS1* and *ALK*, a subset of genes involved in modulating cellular metabolism were found to be dysregulated in NSCLC, including *CYP1B1, GPX7*, *GSTT2* and *BNIP3* (6). Disorders in cellular metabolism have been linked to the pathobiology of several common respiratory diseases and lung cancer (7); aberrantly activated pathways and genes, such as PI3K/Akt/mTOR, RAS/RAF and c-MYC, accelerate glucose and glutamine metabolism to meet the need of energy and building blocks for lung cancer proliferation, while abundant lactate accumulates due to anaerobic glycolysis (8). In recent years, the important pathological functions of lactate have been revealed, it goes in or out of cells dependent on MCT1, MCT4, SLC5A8 and SLC5A12 transporters; it also binds to NDRG3 protein or the membrane receptor GPCR81 to participate in hypoxia response and cellular metabolism, respectively (9, 10). Furthermore, lactate acts as a bona fide agonist to elicit magnesium from endoplasmic reticulum, leading to magnesium uptake by mitochondria and metabolic regulation (11). Through these ways, lactate is able to modulate cellular processes. Lactate was found to fuel TCA cycle in both lung cancer model mouse (12) and human lung cancer patients (13), and cell proliferation was suppressed when lactate utilization was blocked by MCT1 inhibition in lung cancer and colorectal cancer cells (14). Moreover, it was reported that lactate utilization triggered cancer stem cell-like transcriptional profile in human breast cancer cells (15). Recently, histone lactylation was identified as a novel type of epigenetic modification in macrophage, human NSCLC cell line A549 and mouse cells (16, 17), which induced altered gene expression and phenotype in macrophage. Lactate also contributes to the formation of immunosuppression microenvironment (18, 19). These studies demonstrated reprogrammed metabolism as well as the important role of lactate in lung cancer.

However, the regulatory effects of lactate on lung cancer cell metabolic processes are rarely investigated. In this study, we discovered that lactate inhibited glucose uptake and glycolysis while it maintained mitochondrial homeostasis in non-small-cell lung cancer cells. These effects are mainly mediated by altered expression levels of metabolic enzymes due to histone lactylation of the gene promoter.

## Materials and Methods

### Instruments and Reagents

The main instruments in this study included flow cytometer (FACS Celesta, BD, USA), fluorescent quantitative analysis system (Tissue FAXS-S Plus, Tissue Gnostics, Austria), cellular metabolism analysis system (Seahorse XFe96 Analyzer, Agilent, USA), Cytation 5 Cell Imaging Multi-Mode Reader (BioTek, USA), holographic tomographic microscopy 3D cell explorer (Nanolive, Switzerland) and real-time PCR instrument (Light Cycler 480 II, Roche, Switzerland). Antibodies against the following proteins were: G6PD (ab210702), SDHA (ab14715), histone H3 (ab12079) (all from Abcam, Cambridge, MA, USA). Anti-lactyllysine rabbit pAb (PTM-1401) and anti-lactyl-histone H4 (Lys8) antibody (PTM-1405) were purchased from Hangzhou PTM BIO, Co., LTD in China. Alexa Fluor 488 (ZF-0511) and Alexa Fluor 594 (ZF-0513) fluorescent secondary antibodies were purchased from ZSGB-BIO (Beijing, China). XF Cell Mito Stress Test kit (103015-100) and XF Glycolysis Stress Test kit (103020-100) were from Agilent Technologies Inc., and L- (+) lactic acid (L6402) was purchased from Sigma-Aldrich (St. Louis, MO, USA). SYBR- Green (0491850001) and mitochondrial membrane potential assay kit (551302) were respectively from Roche (Palo Alto, CA, USA) and BD (Becton, Dickinson and Company, USA). MitoTracker^®^ Deep Red FAM (M22426) and 6-NBDG (N23106) for glucose-uptake assay were from Thermo Fisher Scientific (Waltham, MA, USA). Cell-light™ EdU Apollo567 In Vitro Kit (C10310–1) was purchased from Guangzhou RiboBio Co., LTD in China. Hoechst33258 (IH0060) was purchased from Solarbio (Beijing, China), and Lactic Acid assay kit (A019-2) was purchased from Nanjing Jiancheng Bioengineering Institute in China.

### Immunofluorescence

Clinical NSCLC samples were obtained from the Affiliated Hospital of Qinghai University. G6PD and SDHA were detected *via* immunofluorescence, with Alexa Fluor 488 (ZF-0511) and Alexa Fluor 594 (ZF-0513) fluorescent secondary antibodies against G6PD rabbit monoclonal antibody (ab210702) and SDHA mouse monoclonal antibody (ab14715), respectively. The dilution ration for the primary antibodies is 100, while that for fluorescent secondary antibodies is 400. Following immunofluorescent staining, the protein levels of G6PD and SDHA were analyzed with Tissue FAXS-S Plus system (Tissue Gnostics, Austria).

### Cell Culture and Treatment With Lactate

The human lung bronchial epithelial cell line BEAS-2B and NSCLC cell lines A549 and H1299 were purchased from the Cell Bank of the Chinese Academy of Sciences (Shanghai, China). The lung bronchial epithelial cells and NSCLC cells were respectively cultured in DMEM/F12 (1:1) and RPMI1640 supplemented with 10% fetal bovine serum at 37°C in a humidified incubator with 5% CO_2_. 1M lactate stock solution was added to the above cell media to reach a final lactate concentration of 5 mM or 10 mM; media with or without lactate were used to culture cells according to experiment requirement under normoxic or hypoxic (1% oxygen) conditions.

### Flow Cytometric Analysis

Control cells and cells treated with 10 mM lactate for 24 hour under normoxic or hypoxic conditions were subjected to flow cytometric analysis, and 5*10^5^ cells were used to perform each assay in triplicate. According to the manufacturer’s instruction, 6-NBDG (N23106, Thermo Fisher Scientific) and mitochondrial membrane potential assay kit (551302, Becton, Dickinson and Company) were respectively utilized to examine the glucose uptake ability and mitochondrial membrane potential of each group of cells treated with different lactate and oxygen concentrations.

### Cell Metabolic Assays

BEAS-2B, A549 and H1299 cells were seeded in XF 96-well and cultured in media with 0, 5 or 10 mM lactate in quadruplicate, and the number of cells per well is 1.5*10^4^; in this way two 96-well plates were prepared and respectively incubated under normoxic and hypoxic conditions for 24 hours prior to cell metabolic analysis. The XF Cell Mito Stress Test kit (Cat# 103015-100) and XF Glycolysis Stress Test kit (Cat# 103020-100) were used following the manufacturer’s instructions to assay glycolysis and mitochondrial metabolism of the cells treated with different lactate and oxygen concentrations. The cells in each well were stained with Hoechst33258 and counted with cytation5; then the raw data were normalized and processed with Wave Software (Version 2.6.1).

### Western Blotting and qPCR

Cultured cells were lysed with RIPA buffer containing proteinase inhibitor to extract total protein, which were quantified using BCA assay, and 20 µg of total protein in each sample was loaded for detection of histone lactylation. For qPCR, total RNA was extracted using TRIzol reagent and 1 μg of total RNA was used for reverse transcription; the reaction system was made according to the manufacturer’s instruction and the assay was run on a Roche Light Cycler 480 II, detection system with the program: pre-degeneration by 95°C for 10 min followed with 40 cycles of 15 s at 95°C and 34 s at 60°C. The mitochondrial gene *mt-Cytb* (NC_001665.2) was cloned into pEASY-T1 plasmid vector to generate pEASY-T1- Cytb, which was then used as standard sample to quantify the mitochondrial DAN (mtDNA) copy number; a series of standard samples containing 0, 10, 10^2^, 10^3^, 10^4^, 10^5^, 10^6^, 10^7^ copies of pEASY-T1-CYB were used to make standard curve, according to which mtDNA copy number in 50 ng of genomic DNA from each sample was examined. Sequences of primers for detecting each target gene are shown in **Table 1**.

**Table 1:** Primer sequences for qPCR.

gene	Forward primer	Reverse primer
HK-1	CTGCTGGTGAAAATCCGTAGTGG	GTCCAAGAAGTCAGAGATGCAGG
G6PD	CTGTTCCGTGAGGACCAGATCT	TGAAGGTGAGGATAACGCAGGC
PKM	ATGGCTGACACATTCCTGGAGC	CCTTCAACGTCTCCACTGATCG
IDH3G	CCAGTGGACTTTGAAGAGGTGC	TTTGTGCGACGGTGGCAGGTTA
SDHA	GAGATGTGGTGTCTCGGTCCAT	GCTGTCTCTGAAATGCCAGGCA
B-actin	GAAGATCAAGATCATTGCTCCT	TACTCCTGCTTGCTGATCCA

### Cell Proliferation Assay

10^4^ cells per well were seeded in 96-well plate and treated with or without lactate (10 mM) in triplicate for 24 hours. Then the cells were incubated in media containing EdU for 2 hours, followed by staining with Apollo fluorochrome according to manufacturer’s instruction. Subsequently, cells were photographed and analyzed with Cytation 5 Cell Imaging Multi-Mode Reader. In addition, cells treated with or without lactate were continuously observed for 6 hours using holographic tomographic microscopy 3D cell explorer to examine cell division. In addition, proliferation and migration of cells treated with or without lactate (5 mM, 10 mM) in triplicate were examined *via* xCELLigence Real Time Cellular Analysis using RTCA DP instrument (Agilent, USA); 5*10^4^ and 1.5*10^4^ cells were seeded per well for proliferation and migration examination, respectively.

### Lactate Measurement in Cell Culture Supernatant

BEAS-2B, A549 and H1299 were seeded in 12-well plate in triplicate; the number of cells in each well is 5*10^4^. Two 12-well plates prepared in the same way were incubated under normoxic and hypoxic conditions for 48 hours. Then Lactate concentration in culture supernatant of each cell was detected following the manufacturer’s instruction.

### Chromatin Immunoprecipitation (ChIP) Assay

A549 cells were cross-linked with 1% (v/v) formaldehyde in phosphate-buffered saline for 10 min at 37°C with gentle shaking. After adding 0.125 M glycine to terminate the reaction, the cells were lysed with lysis buffer on ice. Chromatin DNA was sheared by sonication to obtain ~ 500 bp fragments that were then mixed with anti-lactyl-histone H4 (Lys8) antibody and protein G-agarose to enrich DNA fragments bound to lactylated histone H4 through immunoprecipitation. After decrosslinking, the precipitated DNA was analyzed by qPCR to assess the genomic DNA sequences in *HK-1* and *IDH3G* promoters. Sequences of primers for detecting each promoter site are shown in **Table 2**.

**Table 2:** Primer sequences for ChIP-PCR.

Gene promoter	Forward primer	Reverse primer
HK-1 site 1	ATGTTTGGCAGGTTAGGGAG	TCTGGAGTTCTGGTTCTTGTTC
HK-1 site 2	TGCCCTGACTTGTCTCAAAC	GGTAAATCTAGGACCTGTTCACAG
IDH3G site 1	TCTGGAGTCCGATTGGCTAG	CAATCCCCTCAGTGACAGC
IDH3G site 2	ATTGTGACGTCTCTGGCAG	GGTCGCACCGATTCACGG

### Statistical Analysis

Quantitative data are presented as mean ± SD of at least three experiments. Differences between groups were assessed with the Student’s t-test or by one-way analysis of variance and were considered statistically significant at p<0.05 and highly significant at p<0.01. Data were analyzed using SPSS v.17.0 software (SPSS Inc., Chicago, IL, USA). Survival analysis was performed using Kaplan-Meier analysis via “survival” and “survminer” R packages.

## Results

### NSCLC Presents Altered Expression of Metabolic Enzymes With Aberrant Lactate Metabolism Being Associated With Poor Prognosis

**Figure 1:**
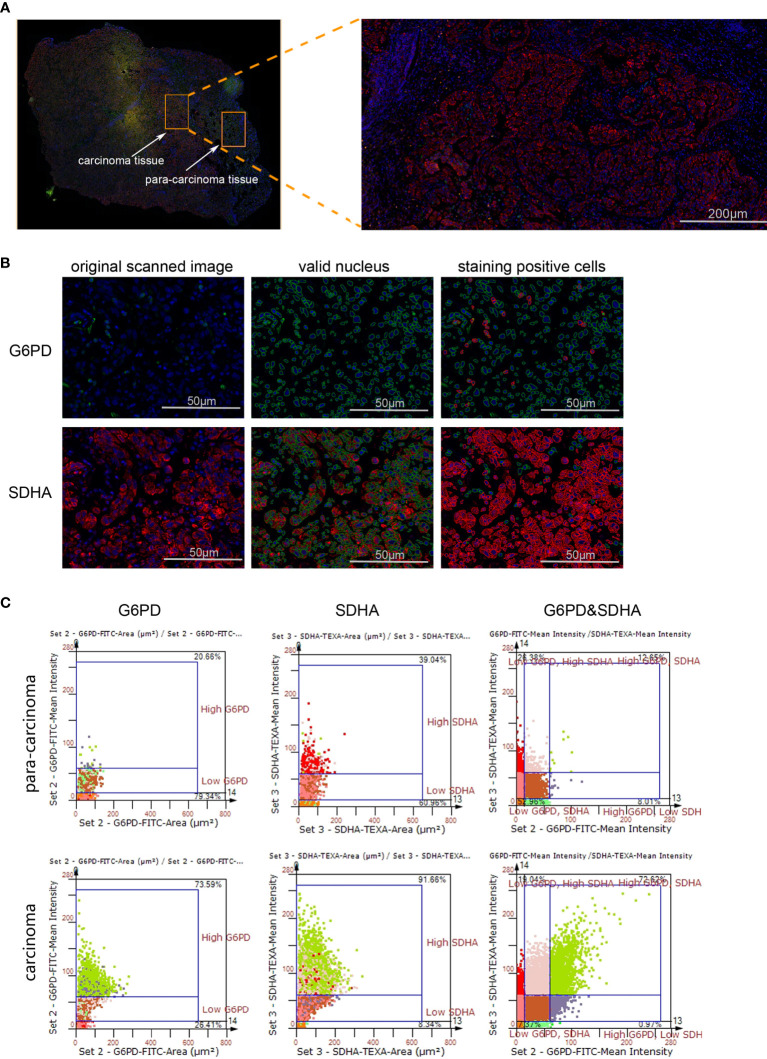
The expression levels of G6PD and SDHA are up-regulated in NSCLC. **(A)** Scanned panorama of human NSCLC tissue stained for G6PD (FITC), SDHA (TEXAS RED) and nuclear DAPI, with the representative carcinoma region amplified. **(B)** Analysis procedure for expression level of target proteins: Valid nuclei, indicated as green circles, were recognized based on size and DAPI intensity; then staining positive cells were identified as indicated by red circles. **(C)** Presentation of G6PD and SDHA expression levels based on staining area and intensity in carcinoma versus para-carcinoma regions. The upper number and lower number in each chart respectively denoted staining positive cells and staining negative ones.

**Figure 2:**
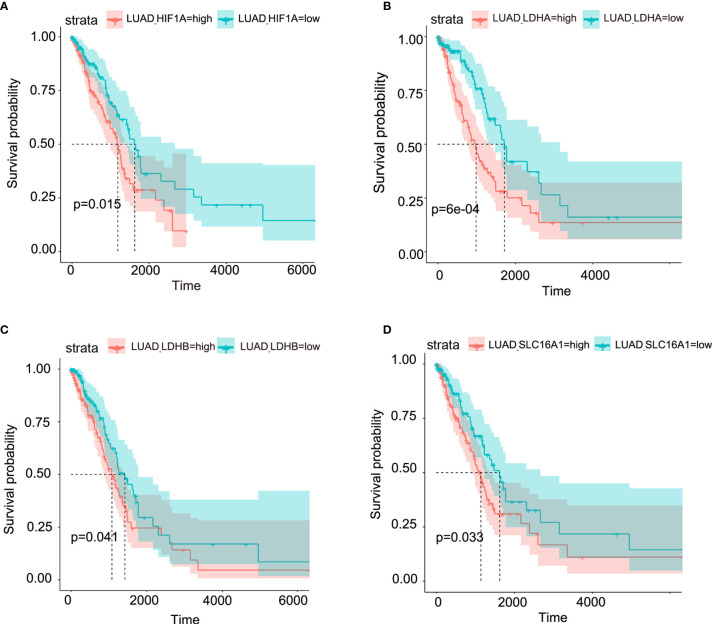
Aberrant lactate metabolism may contribute to human lung adenocarcinoma progression. Elevated expression levels of HIF-1A **(A)**, LDHA **(B)**, LDHB **(C)** and SLC16A1 (encoding MCT1, which can act as lactate transporter) **(D)** are all significantly associated with poor prognosis in human lung adenocarcinoma.

Though metabolic disorders mainly demonstrated as altered nutrient uptake and utilization in lung cancer cells have been widely reported, the alteration in metabolic enzymes was much less investigated. In this study, we examined the expression level of glucose 6-phosphate dehydrogenase (G6PD) and succinate dehydrogenase (SDH) in NSCLC *via* immunofluorescence assay; the examined metabolic enzymes, respectively involved in pentose phosphate pathway and mitochondrial function, displayed higher protein level in cancerous tissues than in paired para-carcinoma tissues (**Figure 1**). In addition, our analysis of TCGA data on lung adenocarcinoma revealed that elevated expression of hypoxia-inducible factor 1A (*HIF1A*) (**Figure 2A**), lactate dehydrogenase A (*LDHA)* (**Figure 2B**), lactate dehydrogenase B *(LDHB)* (**Figure 2C**) and *SLC16A1* (**Figure 2D**) is significantly correlated with poor prognosis. While LDHA and LDHB directly regulate lactate metabolism, HIF1A can stimulate glycolysis (20) and MCT1 encoded by *SLC16A1* can function as lactate transporter (9). These results revealed an increased expression level of metabolic enzymes and the correlation between aberrant lactate metabolism with poor prognosis in lung adenocarcinoma, strongly implying disordered cellular metabolism and an important role of lactate in NSCLC.

### Lactate Inhibits Glycolysis and Maintains Mitochondrial Homeostasis in NSCLC Cells

**Figure 3:**
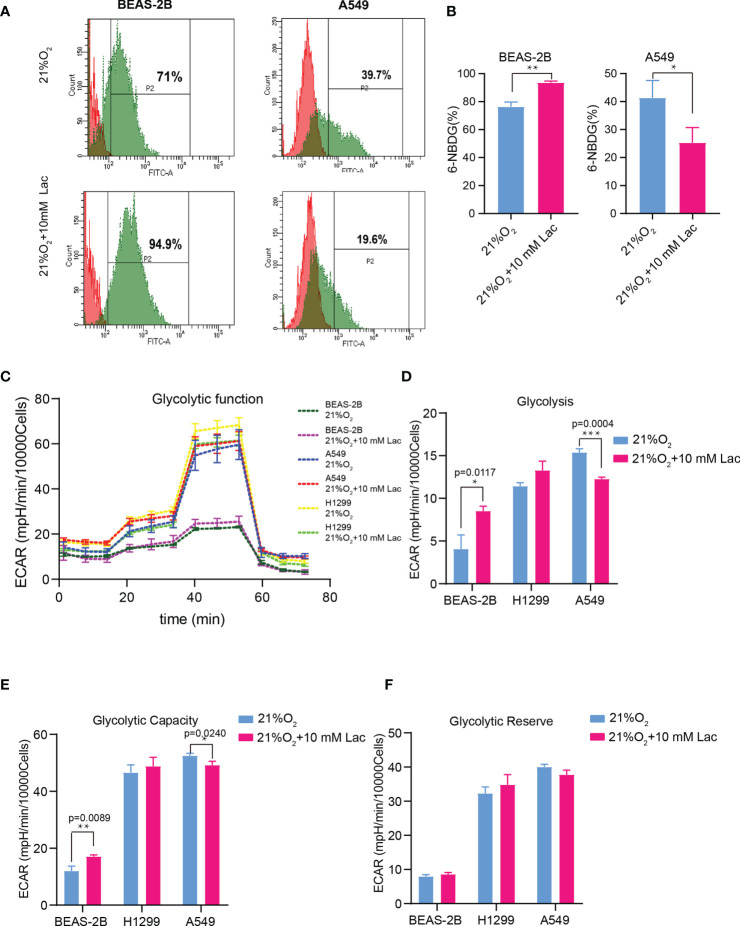
Lactate exerted an inhibitory effect on glucose uptake and glycolysis in NSCLC cells. **(A)** Representative histograms showing distribution of FITC (6-NBDG) intensity among the indicated groups. The number in each histogram denotes the percentage of cells in “P2” that ingested 6-NBDG. **(B)** Comparison of glucose-uptake capability between lactate-treated cells and control ones, indicated as the percentage of 6-NBDG positive cells. **(C)** Glycolytic function curves from glycolysis stress test of BEAS-2B and A549 cells treated with or without lactate under normoxia. Based on glycolytic function curve, glycolysis level **(D)**, glycolytic capacity **(E)** and glycolytic reserve **(F)** of BEAS-2B and A549 treated with or without lactate were analyzed. (*p<0.05, **p<0.01, ***p<0.001).

**Figure 4:**
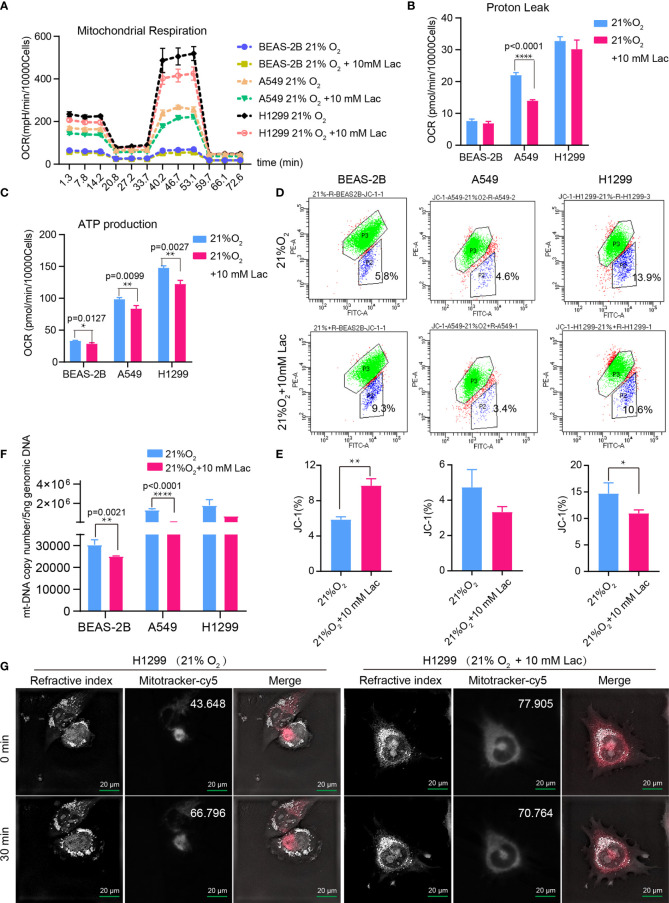
Lactate involves in maintaining mitochondrial homeostasis in NSCLC cells. The cell mito stress test **(A)** was used to determine proton leak **(B)** and ATP production **(C)** levels in the indicated groups of cells *via* cell mito stress test. **(D)** Flow cytometric analysis of mitochondrial membrane potential of BEAS-2B, A549 and H1299 treated with indicated lactate concentration. Cells in “P2” were those with reduced mitochondrial membrane potential. **(E)** Percentage of cells with reduced mitochondrial membrane potential among BEAS-2B, A549 and H1299 cells treated with or without lactate. **(F)** Quantification of mt-Cytb gene copy number in 5 ng total DNA extracted from each of the indicated groups of cells. **(G)** Mitochondria stained with MitoTracker in H1299 cells treated with or without lactate under normoxia; number at the top-right corner denotes mean fluorescence intensity of MitoTracker-cy5. The images were photographed and processed with holographic tomographic microscopy 3D cell explorer. (*p<0.05, **p<0.01, ****p<0.0001).

Based on the above results and the shortage of reported function of lactate in cellular metabolism, the lactate regulatory effects on glucose metabolism were analyzed, which is one of the principal nutrients for mammalian cells. The flow cytometric analysis showed decreased glucose uptake in lactate-treated A549 cells compared with the control group, while BEAS-2B presented increased glucose uptake when treated with lactate (**Figures 3A, B**), implying some difference in metabolic features between lung bronchial epithelial cells and NSCLC cells. In the following investigation of how lactate influenced glycolysis and mitochondrial metabolism *via* Seahorse XFe Analyzer (**Figure 3C**), glycolysis (**Figure 3D**) and glycolytic capacity (**Figure 3E**) was dampened in BEAS-2B and A549 cells treated with lactate, while the effect of lactate on glycolytic reserve was not detected (**Figure 3F**). In Mito Stress Test (**Figure 4A**), both proton leak (**Figure 4B**) and ATP production (**Figure 4C**) were also observed to reduce in mitochondrial metabolism when the cells were treated with lactate. We presumed that the reduced proton leak is due to improved integrity of mitochondrial inner membrane after lactate treatment, and this was supported by the observation that the percentage of cells with decreased mitochondrial membrane potential fell in A549 and H1299 cells when they were treated with lactate (**Figures 4D, E**); however, lactate induced a decrease in mitochondrial membrane potential of BEAS-2B cell. The declined ATP production was likely supposed to result from reduced mitochondrial biomass, as demonstrated by the smaller mtDNA copy number (**Figure 4F**) and weaker mean fluorescence intensity (77.905 versus 70.764) (**Figure 4G**) when the cells were treated with lactate. It is also possible that lactate induced orchestration in mitochondrial function to produce more building blocks than energy for the cells. So lactate reduced the biomass but improved the membrane potential of mitochondria. Taken together, these results indicated that lactate plays a role in inhibiting glycolysis and maintaining mitochondrial homeostasis in NSCLC cells.

### Lactate Modulates Proliferation and Migration of NSCLC Cells

**Figure 5:**
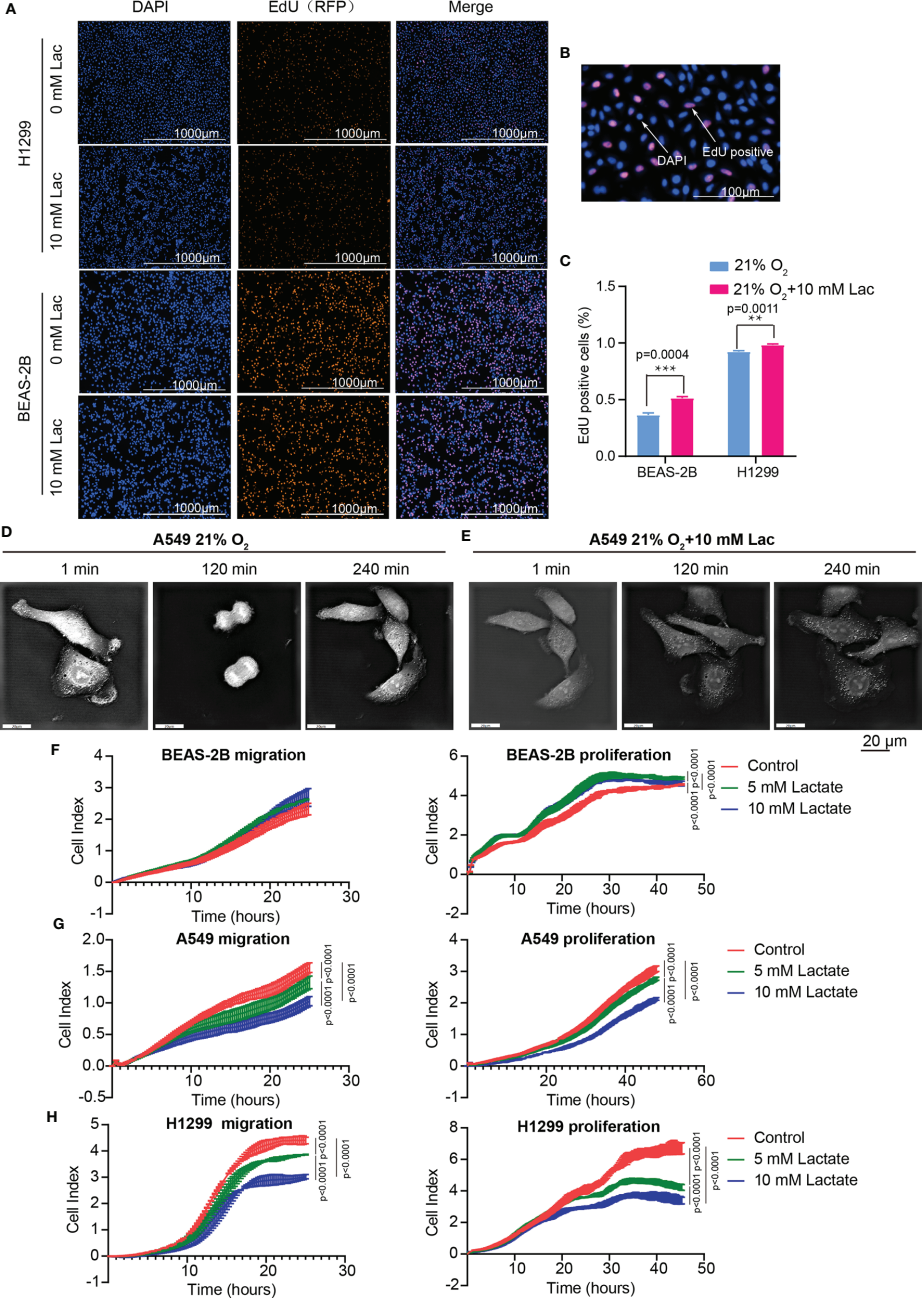
Lactate modulated cell proliferation. **(A)** Representative cell images stained with nuclear DAPI and EdU (RFP) in EdU-incorporating assay. **(B)** Locally amplified image to clearly show EdU-positive cells, which were going through S phase of cell cycle. **(C)** The percentage of EdU-positive cells in the indicated groups. **(D, E)** Cell division progression of A549 cells observed *via* holographic tomographic microscopy 3D cell explorer for 6 hours, when the cells treated without **(D)** or with **(E)** lactate. **(F–H)** RTCA assays of cell migration and proliferation. Migration and proliferation of BEAS-2B **(F)**, A549 **(G)** and H1299 **(H)** when the cells were treated with the indicate concentrations of lactate. (**p<0.01, ***p<0.001, ****p<0.0001).

The effects of lactate on biological properties of NSCLC cells were detected *via* EdU incorporation assay. A larger proportion of EdU-positive cells going through S phase appeared in lactate-treated BEAS-2B and H1299 compared with the control groups (**Figures 5A–C**). In comparison to normal A549 cells (**Figure 5D**), lactate-treated A549 cells spent more time completing cell division, indicating that lactate slows down cell cycle progression of A549 cells (**Figure 5E**). In RTCA assay, while lactate had no effects on migration but promoted proliferation of BEAS-2B cell (**Figure 5F**), both migration and proliferation of A549 (**Figure 5G**) and H1299 (**Figure 5H**) cells were inhibited by lactate. The larger proportion of EdU-positive cells in lactate-treated H1299 cells is probably due to some retardation in S or the following phases. Therefore, lactate also plays a role in modulating cell cycle progression in NSCLC cells.

### Lactate Is Involved in Modulating the Biological Properties of NSCLC Cells Under Conditions That Mimic a Tumor’s Internal Hypoxic Environment

**Figure 6:**
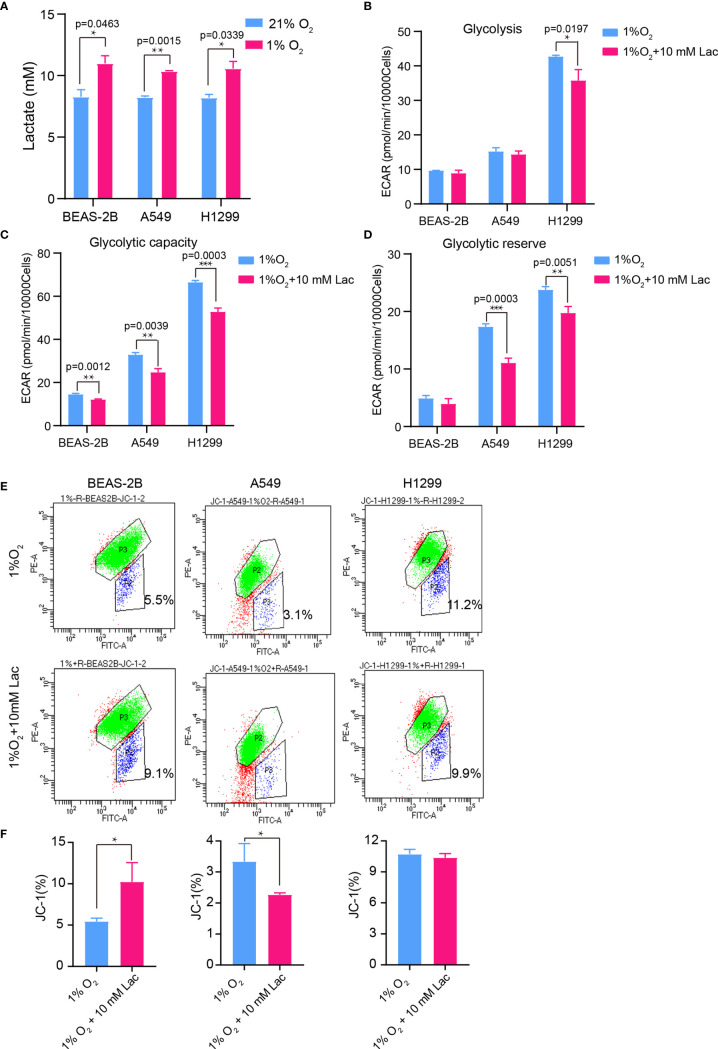
The glycolysis-inhibiting and mitochondrial homeostasis-maintaining effects were also observed under hypoxia. **(A)** Lactate concentration in culture supernatant of BEAS-2B, A549 and H1299 cells under normoxia (21% O2) and hypoxia (1% O2), respectively. Glycolysis **(B)**, glycolytic capacity **(C)** and glycolytic reserve **(D)** of BEAS-2B, A549 and H1299 cells treated with different concentrations of lactate under hypoxia. **(E)** Mitochondrial membrane potential of the indicated groups of cells assayed with flow cytometry. Cells in “P2” were those with reduced mitochondrial membrane potential. **(F)** Percentage of cells with reduced mitochondrial membrane potential among BEAS-2B, A549 and H1299 cells treated with or without lactate under hypoxia. (*p<0.05, **p<0.01, ***p<0.001).

Hypoxia is a hallmark of the interior of solid tumors (21), and is important to remodel cancer cell metabolism, generally resulting in lactate accumulation. To mimic the *in vivo* hypoxic environment, the NSCLC cells were cultured under hypoxic condition (1% oxygen) for 48 hours, and a higher lactate concentration was observed within the culture supernatant under hypoxia than that under normoxic condition (**Figure 6A**). Then lactate concentration was further elevated through addition of exogenous lactate to culture supernatant of NSCLC cells under hypoxia, and their metabolism were compared with that of the non-lactate-treated NSCLC cells under hypoxia. Consistent with the previous results, the metabolic level of glycolysis fell (**Figures 6B–D**), while the mitochondrial membrane potential was maintained in lactate-treated cells under hypoxia (**Figures 6E, F**). So, lactate was able to function as metabolic modulator under both normoxic and hypoxic conditions.

### Histone Lactylation Regulates Expression of Genes Involved in Cellular Metabolism

**Figure 7:**
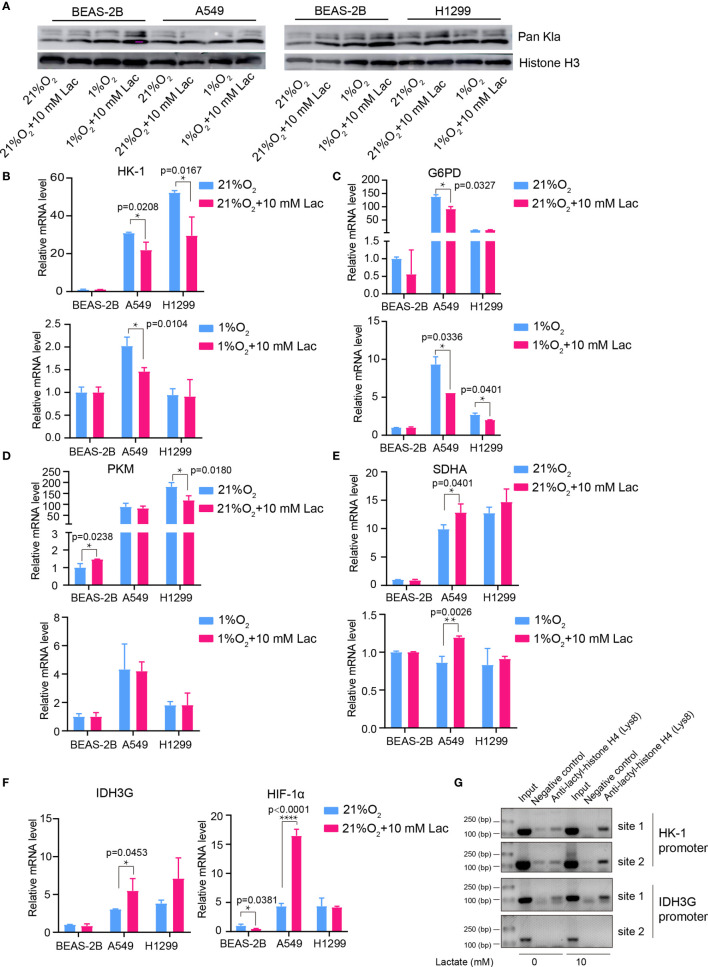
Lactate regulated gene expression through histone lactylation. **(A)** Pan Kla detection in cells treated with different concentrations of lactate under both normoxia and hypoxia. **(B–F)** mRNA levels of metabolic enzymes and HIF1A. HK-1 **(B)**, mRNA levels of G6PD **(C)**, PKM **(D)**, SDHA **(E)**, IDH3G and HIF1A **(F)** in BEAS-2B, A549 and H1299 cells treated with the indicated conditions. **(G)** ChIP assay to detect histone lactylation level in HK-1 and IDH3G promoters. (*p<0.05, **p<0.01, ****p<0.0001).

Given the latest studies reporting lactate as a modulator of gene transcription through histone lactylation (16, 17), it was speculated that the effects of lactate on both cellular metabolism and biological properties in NSCLC cells may result from altered gene expression of critical metabolic enzymes or other factors mediated by histone lactylation. As we expected, when NSCLC cells were treated with lactate, histone lactylation level increased (**Figure 7A**), along with down-regulated transcription of *HK-1* (**Figure 7B**), *G6PD* (**Figure 7C**) and *PKM* (**Figure 7D**) as well as up-regulated transcription of *SDH* (**Figure 7E**) *IDH* and *HIF1A* (**Figure 7F**). Then ChIP assay using anti-lactylated histone H4 antibody was carried out to confirm lactylation of histone lactylation in *HK-1* and *IDH3G* promoters; more promoter DNA sequences of *HK-1* and *IDH* were enriched in the ChIP assay when A549 was treated with lactate (**Figure 7G**), indicating increased histone lactylation in *HK-1* and *IDH* promoters by lactate. Taken together, these results demonstrated that the regulatory effects of lactate on NSCLC cells were probably mediated by lactate-induced promoter histone lactylation of associated genes.

## Discussion

Our findings demonstrated disordered metabolism in NSCLC, and that the metabolite lactate played an important role in modulating glycolysis, mitochondrial homeostasis as well as cell proliferation in NSCLC through histone lactylation-mediated expression of related genes. Disordered metabolism in lung cancer has been well established by remarkably altered metabolism of substances including lipid and glutamine (22, 23). In our study, the metabolic disorder in NSCLC was demonstrated by elevated expression levels of *G6PD* and *SDHA* in carcinoma tissues than in paired para-carcinoma tissues (**Figure 1**). In addition, compared with the human lung bronchial epithelial cell line BEAS-2B, the human NSCLC cell lines A549 and H1299 presented higher basal levels of glycolysis (**Figures 3D, E**) and metabolic enzymes (**Figures 7B–F**); lactate also induces disparate alterations in glucose uptake (**Figures 3A, B**) and mitochondrial membrane potential (**Figures 4D, E**) between BEAS-2B and NSCLC cells. These observations demonstrated that NSCLC cells possess a quite distinct metabolic status from that of normal cells, and the specific metabolic features may contribute to better tumor classification and discovery of therapeutic target (24).

Disordered metabolism in cancer is generally caused by hypoxia in interior of cancers and genetic mutations (21, 25, 26); however, the cellular metabolism is not passively affected, but actively reprogrammed to survive the harsh environment. Accumulated lactate resulting from reprogrammed metabolism can refuel TCA cycle in NSCLC (12, 13). Our study discovered that lactate attenuates glycolysis (**Figure 3**) while maintaining mitochondrial homeostasis (**Figure 4**) in NSCLC cells, and expression levels of the analyzed metabolic enzymes in glycolysis, pentose phosphate pathway and TCA cycle are respectively down-regulated and up-regulated by lactate (**Figure 7**). So TCA cycle is the preferential metabolic pathway in response to lactate in NSCLC cells. In human NSCLC, glucose is the main nutrient metabolized in less perfused regions, while highly perfused regions mainly utilize non-glucose nutrient, including lactate (27). Therefore, lactate probably mediates the symbiosis between less perfused and highly perfused regions, contributing to tumor progression. Furthermore, the involvement of lactate in modulating cellular metabolism is demonstrated by lactate-induced transcriptional activation of *HIF1A* in A549 cell (**Figure 7F**), and this effect of lactate on *HIF1A* transcription was also recently observed in human MCF7 breast cancer cell (28). HIF-1 can in turn activate transcription of multiple genes, including *SLC2A1*, *SLC2A3*, *LDHA* and *PDK1*, to facilitate glucose uptake and glycolysis (20), resulting in lactate generation. So there likely exists positive feedback between lactate and HIF-1 in reprogramming cancer cell metabolism.

Consistent with the effects of lactate on glycolysis and mitochondrial function, the mRNA levels of metabolic enzymes like *HK-1* and *PKM* in glycolysis as well as *SDHA* and *IDH3G* in TCA cycle were respectively down- and up-regulated by lactate (**Figures 7B–F**). This may involve increased histone lactylation in *HK-1* and *IDH3G* promoters (**Figure 7G**).

Based on these results, it can be concluded that lactate promotes cell proliferation and modulates cellular metabolism at least in part through histone lactylation-mediated gene expression in non-small cell lung cancer cells. But further investigation is needed to elucidate why histone lactylation is associated with both up-regulation and down-regulation of gene transcription.

## Data Availability Statement

The original contributions presented in the study are included in the article/supplementary material. Further inquiries can be directed to the corresponding author.

## Ethics Statement

The study protocol was performed in accordance with the guidelines outlined in the Declaration of Helsinki. The Ethics Committee of Affiliated Hospital of Qinghai University approved the study.

## Author Contributions

YM and DH: conceptualization. YM and JJ: funding acquisition, project administration and manuscript revision. YJ and JH: methodology. MT, JL, and YZ: data curation. TZ, ZhiL, ZhoL, and ST: software. DH: writing. All authors contributed to the article and approved the submitted version.

## Funding

This work was supported in part by grants from the Science and Technology Agency of Qinghai Province (2017-ZJ-710), Qinghai Health Committee (No. 2020-wjzd-03) and Qinghai University (2020-QYY-5).

## Conflict of Interest

The authors declare that the research was conducted in the absence of any commercial or financial relationships that could be construed as a potential conflict of interest.

## Abbreviations

chromatin immunoprecipitation (ChIP), glucose 6-phosphate dehydrogenase (G6PD), hypoxia-inducible factor 1A (HIF1A), lactate dehydrogenase (LDH), mitochondrial DAN (mtDNA), non-small-cell lung cancer (NSCLC), small-cell lung cancer (SCLC), succinate dehydrogenase (SDH)
